# Practical ways to deal with the high burden of cardiovascular disease in hemodialysis patients

**DOI:** 10.1590/S1516-31802006000100008

**Published:** 2006-01-05

**Authors:** José Jayme Galvão de Lima

**Keywords:** Renal dialysis, Chronic kidney failure, Cardiovascular diseases, Risk, Uremia, Diagnosis, Hemodiálise, Insuficiência renal crônica, Doenças cardiovasculares, Risco, Uremia, Diagnóstico

## Abstract

Cardiovascular disease is the main cause of death among hemodialysis patients. Although uremia by itself may be considered to be a cardiovascular risk factor, a significant proportion of dialysis patients die because of cardiovascular disease not directly attributable to uremia. Indeed, many of the cardiovascular diseases and cardiovascular risk factors in these patients are common to those occurring in the general population and are amenable to intervention. Lack of proper medical care during the early stages of renal insufficiency and present-day dialysis routines, by failing to correct hypertension, hypervolemia and left ventricular hypertrophy in many patients, may also add to the cardiovascular burden. The author suggests that, in addition to early treatment and referral to a specialist, chronic renal failure patients should undergo intensive cardiovascular screening and treatment, and correction of cardiovascular risk factors based on guidelines established for the general population.

## RESUMO

**CONTEXTO:**

A doença cardiovascular é a principal causa de morte em pacientes tratados por hemodiálise crônica. Embora a uremia por si só possa ser considerada um fator de risco cardiovascular, uma proporção significativa de pacientes tratados por diálise morre devido a problemas cardiovasculares não diretamente atribuíveis à uremia. De fato, muitas das alterações cardiovasculares e fatores de risco cardiovascular observados em pacientes renais crônicos são comuns aos que ocorrem na população geral e podem ser controlados por medidas de comprovada eficácia em indivíduos não-doentes renais. A falta de cuidados médicos apropriados durante as fases iniciais da insuficiência renal e os atuais métodos e rotinas empregadas na diálise, por não conseguirem garantir o controle adequado da hipertensão, hipervolemia e da hipertrofia ventricular, em muitos pacientes, contribuem para o aumento da pletora de problemas cardiovasculares. O autor sugere que, em adição à instituição de tratamento adequado e envio precoce aos especialistas, doentes renais crônicos devam ser submetidos a avaliação cardiovascular minuciosa visando o tratamento das alterações cardiovasculares e correção dos fatores de risco baseados nas diretivas estabelecidas para a população geral.

## INTRODUCTION

Cardiovascular disease (CVD) is prevalent in patients with renal failure and accounts for 50% of all dialysis deaths.^1^ Raw (nonadjusted) mortality rates in industrialized countries during the first year on hemodialysis range from roughly 10% in Western Europe and Japan to 20% in the United States.^1,2^ The corresponding figure in Brazil is 18%.^2^ Estimates from different countries indicate that more than 60% of patients die before completing 10 years on dialysis.^3^ This high mortality rate is at least in part caused by the extraordinarily high frequency of cardiovascular risk factors and established CVD that are present in a significant proportion of these patients. However, a close look at the extensive list of cardiovascular risk factors and CVD associated with chronic uremia shows that many risk factors are preventable or amenable to control or attenuation by appropriate therapeutic interventions. The author therefore hypothesizes that the high morbidity and mortality rates observed among kidney failure patients might, in some cases, be a consequence of underdiagnosis and underutilization of interventions that have proven useful among the general population for preventing and treating CVD. The author also believes that dialysis, in the way it is performed nowadays, may contribute towards mortality by failing to correct for or prevent important cardiovascular risk factors.

The present discussion makes use of information from the literature and from personal experience to suggest some practical ways for preventing and reducing cardiovascular complications due to dialysis. The personal contribution is based on data from observations of two populations of hemodialysis patients followed up at the author's institution during the last 10 years: a low-risk group of 120 relatively young patients with low prevalence of comorbidity and a high-risk cohort of 335 subjects who were at least 50 years old and presented diabetes or clinically evident CVD, or both of these (Table 1).^4-7^

**Table 1:** Characteristics of low and high-risk hemodialysis patients followed up at Instituto do Coração (InCor)^4-7^

Characteristics	Low-risk	High-risk
**n**	103	335
**Follow-up range (months)**	6-120	2-84
**Time on dialysis (months)**	30 (median)	38 (median)
**Age (years)**	44 ± 13	56 ± 8
**Males**	50%	72%
**Whites**	73%	66%
**Smoking**	17%	27%
**Diabetes**	3%	37%
**Dyslipidemia**	28%	43%
**Hypertension**	36%	86%
**Myocardial infarction**	0%	10%
**Myocardial revascularization**	0%	4%
**Stroke**	0%	9%
**Vasculopathy**	0%	28%
**Heart failure**	0%	9%
**CAD (stenosis > 70%)**	Not determined	43%
**LV hypertrophy**	75%	94%

*CAD = coronary artery disease; LV = left ventricular.*

### Identification of high-risk hemodialysis patients

Prompt identification of high-risk patients will result in more successful interventions. This is accomplished by identifying individuals with established CVD or those at risk of developing CVD, and calculating the probability of developing cardiovascular events in the future. Among the general population, the latter is usually achieved by applying equations derived from prospective studies in large groups of individuals who were followed up for long periods of time, as described below.

### Cardiovascular risk factors

#### Traditional cardiovascular risk factors

Among the general population, cardiovascular risk is usually calculated using the Framingham equations.^8^ These equations should be applied only to individuals without overt cardiovascular target organ damage because, in such cases, by definition, the cardiovascular risk is already greater than 20% over a ten- year period, while a 20% event probability per decade is arbitrarily used to define highrisk status. Variables used in these equations include age, sex, diabetic and smoking status, total and high-density lipoprotein-cholesterol (HDL-cholesterol) and systolic and diastolic blood pressure. Because of the high rate of established cardiovascular disease in end-stage kidney failure, the Framingham equations are considered to be of limited use for calculating the probability of events for the whole population of dialysis patients.^9,10^

The Choice Study^11^ found that the projected five-year cardiovascular risk based on the Framingham Risk Equation was 13% among dialysis patients over 40 years old without overt cardiovascular disease and was 6% among the controls matched for age, sex and race. Cheung et al.^12^ reported no significant difference between the calculated cardiovascular risk among low-risk dialysis patients and age-matched controls.

The present author's group compared the absolute and relative 10-year cardiovascular risks among 123 hemodialysis patients, who had an 86% prevalence of hypertension, with those among an age-matched group of essentially hypertensive subjects who were also followed up at the author's institution. The absolute risk did not differ between the groups and was close to 15%, whereas the relative risk was twice as high as among the low-risk Framingham control population.^13^ In the study by the present author's group, 20% of the dialysis patients and 19% of the hypertensive patients presented a risk of more than 20% per decade of life that they would have the event.^13^ Because the cardiovascular risk in the nonselected dialysis population is 10 to 15 times higher than in the general population,^14^ these results have been interpreted as an indication that the Framingham equations explain only a small part of the extraordinarily high cardiovascular risk among end-stage kidney failure patients.^11,15^

In fact, it seems likely that the patients classified as low-risk may have occult CVD that will become manifest in the future. In agreement with this view, the present author's group observed that 30% of their dialysis patients included in the abovementioned analysis of Framingham Risk Equations presented ≥ 70% coronary stenosis in one or more coronary arteries, in spite of the absence of clinical evidence of coronary artery disease (CAD).^13^ Alternatively, it is also possible that some dialysis patients without overt cardiovascular disease are indeed at relatively low risk of developing cardiovascular complications. Prospective studies are necessary to determine whether this relatively low risk will be confirmed by a comparable low prevalence of events in the future.

### Nontraditional cardiovascular risk factors

Because the classical risk factors included in the Framingham equations fall short in explaining the high prevalence of cardiovascular events among dialysis patients, there has been speculation that this occurs because some alterations that are peculiar to the uremic syndrome are not included in the risk calculations.^10^ Anemia, chronic hypervolemia, underdialysis, abnormal calcium and phosphate metabolism, hyperhomocysteinemia, high lipoprotein(A), oxidative stress and low-grade inflammation could significantly contribute towards the cardiovascular damage in kidney failure. However, except perhaps for anemia, hypervolemia and altered calcium-phosphate metabolism, evidence linking these factors to the prognosis is still fragmentary, and so far this evidence has not been integrated into one comprehensive equation.

### Established cardiovascular diseases

Probably more important than traditional and nontraditional cardiovascular risk factors in determining outcomes for dialysis patients is the existence of established cardiovascular disease. In one large study,^15^ at least half of all the patients starting on dialysis had overt CVD. A history of cardiac failure was present in 35%, coronary disease in 32%, peripheral vascular disease in 17% and cerebrovascular disease in 10% of the cases. Also, left ventricular (LV) hypertrophy, as assessed by echocardiography, attained a frequency close to 80%. In the patient series with prevalent high risk studied by the present author's group (Table 1), 43% had clinical evidence of at least one CVD: heart failure, 9%; peripheral vascular disease, 28%; previous stroke, 9%; history of myocardial infarction, 10%; and history of myocardial revascularization, 4%. LV hypertrophy was observed in 94%. It was also found that the presence of established CVD had a clear impact on the prognosis. After a mean follow-up of six years, the patients with evidence of CVD at the inception had four times more chance of developing cardiovascular events.

### Late referral for dialysis

Patients starting on dialysis without previous medical care have a higher frequency of early death (within one year),^16^ reduced overall survival,^17^ excessive morbidity,^18^ and poorer quality of life.^19^ This is not surprising, considering that many disorders, including CVD, are already present in many patients, from the earliest stages of renal insufficiency. According to Sesso and Belasco,^17^ 58% of the patients admitted to a maintenance dialysis program in Brazil had a late diagnosis. In the present author's group of 335 high-risk patients, 32% of the cases had their diagnosis of renal failure established less than 60 days before starting on dialysis, and only 5% of these patients had access to nephrologists before reaching the final stages of their disease. Lack of proper medical care not only contributes towards accelerating the progression of the renal disease, but it also leaves unchecked many cardiovascular risk factors. Therefore, early diagnosis of kidney failure and referral to a nephrologist may have a significant impact on reducing the CVD burden of these patients.

### Underdialysis

Ideally, good renal replacement therapy would perform like a normal kidney, keeping metabolic and hemodynamic parameters close to normal. If this concept is accepted, it follows that all types of dialysis presently in use provide underdialysis. In fact, many patients dialyzed according to state-of-the-art dialysis techniques are hypertensive and only infrequently achieve a sustained state of euvolemia.^20^ It is therefore conceivable that complications and mortality due to dialysis are related to insufficient and, sometimes, substandard substitutive renal function. Evidence supporting this view is provided by data showing that survival on dialysis is proportional to the dialysis dosage, which is usually calculated in terms of hours of dialysis delivered per week or month or by the fractional clearance of urea (KT/V).^21,22^

Treatment with fewer hours of dialysis than the minimum 12 hours per week recommended is accompanied by higher mortality rates.^23^ For example, a retrospective European study,^23^ including more than 70,000 patients, showed that the gross mortality rate over one year was 8.3% for patients dialyzed for at least 12 hours per week and 11.8% for those dialyzed for 9 to 11 hours per week.

Thus far, the highest long-term hemodialysis survival rates have been reported by groups that use the largest doses and the longest times for dialysis.^24^ In this respect, the duration or frequency of dialysis sessions appears to correlate better with survival than with KT/V.^25-27^ This could be expected, because adequate KT/V does not presuppose control of either hypertension or hypervolemia, among other variables influencing the prognosis. Although economic and logistic difficulties create limitations on the widespread use of longer dialysis times, at least it is important to provide an effective minimum of 12 hours per week, on dialysis schedules with adequate KT/V.

Over the past decade, new hemodialysis options have been introduced and old ones resuscitated with encouraging results, including daily short dialysis and nightly hemodialysis. These approaches appear to provide better control over blood pressure, nutritional state, bone metabolism and anemia than does the traditional method.^28^

### Underprescription of cardioprotective drugs

Because the probability of future cardiac events in patients with chronic renal disease surpasses 20% in ten years, kidney failure is now considered to be a "coronary heart disease equivalent.^29^ Angiotensin-converting enzyme inhibitors (or AT1 blockers), low- dose aspirin, beta blockers and statins reduce the incidence and severity of CVD, and their use is considered mandatory for high-risk patients.^29^ In practical terms, this means the routine prescription of the first three of these drug types for all dialysis patients, and statins for those with increased cholesterol levels. However, compelling evidence indicates that this practice is frequently overlooked.^30-32^ For instance, in one study,^30^ fewer than 50% of the chronic kidney failure patients received each type of agent, even when established CVD was present. Among the present author's high-risk group of hemodialysis patients, it was observed that 74% were taking none or only one of the four cardioprotective drug types.^13^ This situation was not altered when diabetic patients and those with CAD were analyzed separately.

### Coronary artery disease

The importance of CAD with regard to the prognosis for dialysis patients can hardly be exaggerated, because cardiac ischemic events are involved in 40% to 50% of all cardiovascular deaths in this population.^9^ Therefore, early identification and proper interventions will, very likely, have an important effect on the prognosis for patients with CAD. The reported proportions of dialysis patients with CAD varies in relation to many factors like age, diabetic status, length of follow-up and the diligence with which CAD is investigated. The angiographically confirmed prevalence of CAD with stenosis of more than 50% ranges from 24% among low-risk patients to 85% among older patients with diabetes.^33^ However, most of these estimates originated from studies in which angiography was performed only on patients with clinical evidence of CAD or whose noninvasive test results were suggestive of CAD.

Using coronary angiography, the present author's group studied 335 consecutive high-risk renal transplant candidates who were sent to their institution for preoperative evaluation, independently of the symptoms. Coronary stenosis ≥ 40% was found in 60% of the patients and ≥ 70% in 43%. It was also observed that the patients’ angina and noninvasive test results (myocardial scintigraphy and dobutamine-stress echocardiography) had a poor correlation with significant CAD.^4^ Moreover, the presence of stenosis ≥ 70% was the best predictor of cardiovascular events, in comparison with clinical stratification and noninvasive testing. After 48 months, the probability of event-free survival was 94% for patients with stenosis < 70% and 54% for those with stenosis ≥ 70%. Patients with significant CAD had almost ten times higher relative risk of developing events than those with normal coronary arteries or less severe degrees of CAD. Both myocardial scintigraphy and stress echocardiography failed to identify roughly 30% of the patients who developed events. These results suggest that coronary angiography should be performed on all high-risk dialysis patients. We are presently evaluating whether noninvasive testing offers any advantage over clinical stratification among low-risk dialysis patients. Prospective large-scale studies are necessary to define the location for invasive coronary interventions among this population.

### Diabetes

Diabetes is highly prevalent among dialysis patients. Diabetes, age, and CAD are the most important factors influencing survival when on dialysis, and diabetic patients continue to do worse than nondiabetic patients on renal replacement therapy.^26^ Diabetes increases the incidence and severity of cardiac and extracardiac atherosclerosis and is frequently associated with malnutrition. Many centers perform coronary arteriography routinely on diabetic patients with end-stage renal disease prior to kidney transplantation.^34^ Thirty-seven percent of the high-risk patients studied by the present author's group had diabetes (Table 1).^4-7^ Among these patients, significant CAD was observed in 53%, compared with a prevalence of 35% among nondiabetic patients (p < 0.05). One reasonable speculation is that diabetic patients have more cardiovascular events because they present higher prevalence of CAD, but this may be only partially true. The present author's group observed that patients with diabetes without CAD presented a risk of developing major cardiovascular events that was similar to that of nondiabetic patients with CAD.^5^ Over a six-year period, the event-free survival rate was comparable between the two groups and less than 50% for both of them. On the other hand, subjects without either disease presented an event-free survival rate of close to 85% over the same observation period. These results indicate that diabetes confers a cardiovascular risk similar to that of CAD and points towards the need for intensive treatment of diabetes and its complications. They also suggest that coronary angiography should be performed on all diabetic patients undergoing dialysis, independently of symptoms and the possibility of kidney transplantation.

### Hypertension

Hypertension is probably the most frequent cardiovascular risk factor found among dialysis patients, but its prevalence is highly variable. In general terms, the prevalence of hypertension in nonselected dialysis populations is usually greater than 70%.^35^ In a recent report by Agarwal et al.,^36^ hypertension was documented in 86% of 2,535 patients and was adequately controlled in only 30%. In the cohort of 335 high-risk patients studied by the present author's group, 86% were hypertensive and an additional 9% were normotensive with the use of drugs at the initial evaluation. On the other hand, the prevalence of noncontrolled hypertension was 36% of 103 younger, low-risk subjects also studied by the present author^37^ (Table 1). It is worth mentioning that more than 90% of the patients with hypertension in both populations were taking antihypertensive medication. This high rate of treatment failure is likely to be influenced by a state of chronic hypervolemia, due to insufficient fluid removal by dialysis, as discussed above. Control of blood pressure through normalization of extracellular volume is feasible, but usually requires prolonged duration of dialysis or more frequent dialysis sessions.^37^ In addition, the present author's group has observed that nephrologists tend to tolerate some degree of hypertension in their patients, with the objective of reducing the episodes of hypotension induced by fluid removal during dialysis.^37^ All this results in blood pressure levels far above the recommended target level of 140/80 mmHg for patients with chronic uremia.^9^ Hypertension might exert a number of negative effects, including left ventricular hypertrophy (LVH), heart failure and CAD. Therefore, adequate blood pressure control must be a major objective in the management of patients on dialysis.^38^ The present author's group found that systolic blood pressure was a strong predictor of complex arrhythmia,^39^ LVH and ventricular remodeling.^37^ Even more importantly, it was also observed that hypertension was associated with reduced long-term survival. The risk of dying was 2.2 times higher among hypertensive patients, and increased by 7% for each additional year of age and by 0.7 % for each one-gram increase in the left ventricular mass index.^37^

### Left ventricular hypertrophy

Left ventricular hypertrophy (LVH) is also highly prevalent and a major predictor of death among dialysis patients.^37,40^ The causes of LVH include age, hypertension, hypervolemia, anemia and arterial-venous fistula for dialysis access. LVH (defined as left ventricular mass index, LVMI > 125 g/m^2^) was detected in 94% and 75% of the high and low-risk groups studied by the present author's group, respectively, and was influenced by systolic blood pressure and hematocrit level.^41^ In this study, concentric hypertrophy, which is associated with higher cardiovascular risk and target organ damage, in contrast with eccentric hypertrophy,^42^ was restricted to patients with previous or uncontrolled hypertension. In the subgroup of patients who had no past or present hypertension, 61% had normal ventricles, 9% LV concentric remodeling and 30% eccentric hypertrophy.^41,43^ Other studies have shown that regression of LVH in chronic kidney failure cases can be achieved with reduction in blood pressure.^44,45^ The present author's group also observed significant long-term reduction in LVMI with good blood pressure control after kidney transplantation.^46^

### Lipid metabolism

The serum lipid profiles of hemodialysis patients have been extensively studied and typically consist of normal or low total cholesterol and low-density lipoprotein cholesterol (LDL-C), low high-density lipoprotein cholesterol (HDL-C), and moderately increased triglycerides, Lp(a) and intermediate density lipoproteins.^6^ The prevalence of dyslipidemia is influenced by many factors, such as age, nutritional state, target population and comorbidity (especially diabetes). For instance, in the low and high-risk groups studied by the present author's group, the prevalence of dyslipidemia, defined as total cholesterol and/or triglycerides greater than 200 mg/100 ml, was 28% and 43%, respectively (Table 1).^4-7^ The role of dyslipidemia in relation to the cardiac risk of dialysis patients is unclear, because of the presence of multiple confounding factors and the paucity of prospective studies encompassing a large number of individuals. Also, no clear and definitive evidence exists regarding the beneficial effects of lipid-lowering agents on reducing cardiovascular complications. On the other hand, low total cholesterol levels are associated with increased overall and cardiovascular mortality when on dialysis, possibly as a reflection of malnutrition and inflamma- tion.^47^ In spite of this, the present recommendations are to treat individuals with end-stage renal disease as presenting high cardiovascular risk, using the lipid targets for patients with atherosclerosis but no kidney disease.^9^ This means targeting the total cholesterol level at < 200 mg/100 ml and LDL-cholesterol at < 160 mg/100 ml, using drugs as needed. More stringent control of LDL-cholesterol is required for patients with manifest cardiovascular disease, as advised for the nonuremic population. As discussed above, many patients with dyslipidemia are not receiving cholesterol-lowering drugs, despite evidence that such drugs are safe for dialysis patients.

### Malnutrition

Malnutrition is one of the most important causes of death when on dialysis^7,48^ and has been reported in as many as 36% of patients admitted for renal replacement therapy.^49^ Better nutritional status is related to prolonged survival.^50,51^ Malnutrition appears also to correlate with cardiovascular complications and cardiovascular death when on dialysis, although it is not clear whether this reflects a cause-effect relationship. What is evident is that malnutrition markers (low hematocrit levels and reduced serum creatinine, total cholesterol and albumin) and micro-inflammation frequently coexist and are closely related to high levels of atherogenic cardiovascular risk factors and cardiovascular death.^52^ Therefore, identification of patients with malnutrition will also reveal those at greater cardiovascular risk. Statins and angiotensin conversion enzyme (ACE) inhibitors have been shown to reduce C-reactive protein, blood pressure and cholesterol in the general population^53^ but, as discussed above, these medications are not frequently prescribed to dialysis patients.

## CONCLUSIONS

Cardiovascular disease must be actively pursued and treated in all hemodialysis patients. CAD is of special concern because it is frequent, often asymptomatic and is a strong predictor of death. Patients without clinically apparent CVD should be considered to be at high risk and treated as such. All measures proven useful in the general population for preventing and reducing the risk of cardiovascular events must be used, including cardioprotective drugs. Patients with diabetes are particularly prone to events because diabetes entails a risk of events similar to what is associated with CAD. Chronic hypervolemia, hypertension and left ventricular hypertrophy are probably better treated or prevented by providing the best possible quality of dialysis or, even better, extending the duration or frequency of the dialysis sessions. These measures have the highest possibility of success when started during the initial stages of kidney failure, before starting dialysis. Early diagnosis and referral to a specialist are essential for achieving these goals.
